# Foldable Denture: For Microstomia Patient

**DOI:** 10.1155/2012/757025

**Published:** 2012-08-22

**Authors:** Sandeep Kumar, Aman Arora, Reena Yadav

**Affiliations:** Department of Prosthodontics and Crown & Bridge, D.A.V. (C) Dental College & Hospital, Yamunanagar, Haryana 121006, India

## Abstract

Microstomia may result from surgical treatment of orofacial neoplasms, cleft lips, maxillofacial trauma, burns, radiotherapy, or scleroderma. A maximal oral opening that is smaller than the size of a complete denture can make prosthetic treatment challenging. This clinical paper presents the prosthodontic management of a total edentulous patient with microstomia. Sectional mandibular and maxillary trays and foldable mandibular and maxillary denture were fabricated for the total edentulous patient.

## 1. Introduction

It has been reported that the limited oral opening may result from the surgical treatment of orofacial cancers, cleft lips, trauma, burns, Plummer-Vinson syndrome, or scleroderma.

The maximum oral opening that is smaller than the size of complete denture can make the prosthetic treatment challenging. Several techniques have been described for use when either standard impression trays or the denture itself becomes too difficult to place and remove from the mouth.

Sectional dentures have been recommended, with the denture pieces connected by the clasps. McCord et al. [1] describe a maxillary complete denture consisting of 2 pieces joined by a stainless steel rod with a diameter of 1 mm fitted behind the central incisors. Luebke [2] describe a sectional impression procedure for edentulous patient by using 2 plastic sectional impression trays assembled with Lego building blocks and autopolymerizing resin.

In this paper, a different design for the fabrication of maxillary and mandibular sectional trays and a foldable maxillary and mandibular complete denture is described.

## 2. Case Report

**Figure 1:**
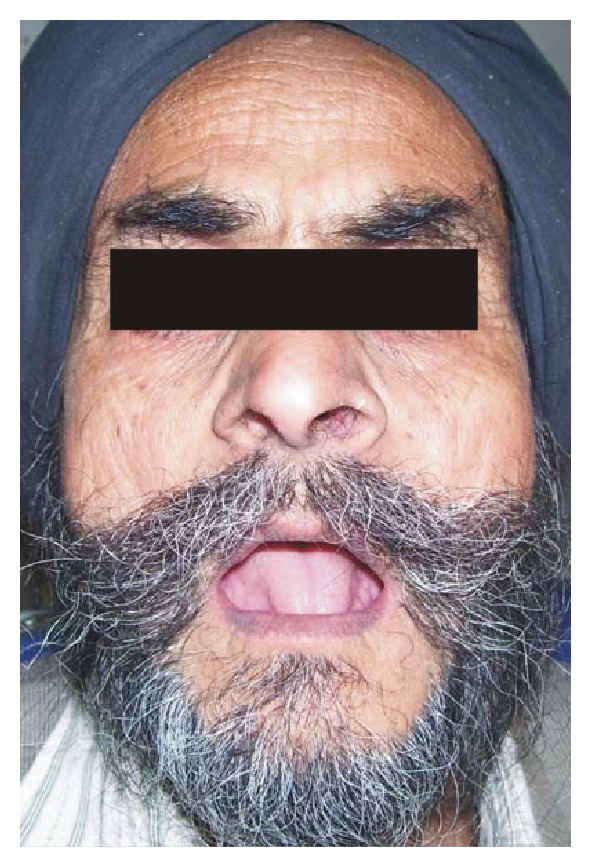
Preoperative photograph.

A 64-year-old edentulous male sought treatment at the Prosthodontic Department in D.A.V. (C) Dental College, Yamunanagar, Haryana. He had a limited oral opening of about 25 mm (Figure 1). There was no suggestive history of smoking, alcoholism, or any other systemic disease. On clinical examination, upper and lower ridges were found to be in favourable condition. Various treatment options were discussed, and the patient accepted the treatment described below.

## 3. Procedure

**Figure 2:**
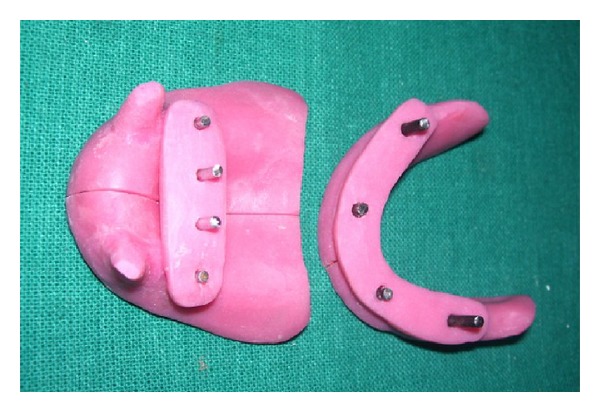
Sectional special tray.

Preliminary impressions for both dental arches were obtained with a putty silicon impression material (Imprint, 3M ESPE, Germany) with the help of finger pressure. The impressions were poured in dental stone (Kalstone, Kalabhai Karson, Mumbai) to obtain primary cast. An autopolymerizing acrylic resin (DPI RR cold cure, DPI, India) tray was prepared on each stone cast. For each tray, 4 metal pins were attached, each of 2.5 mm in diameter; two of these pins were 25 mm long, and the other two were 15 mm long. In mandibular tray, the long pins were placed close to the distal end and the short pins close to the midline and in the maxillary tray, the short pins were placed over the residual ridges and the long pins close to the midline (Figure 2).

The acrylic resin trays were lubricated with petroleum jelly, and an acrylic resin block that slid tightly on the pins was prepared. The trays were cut into two pieces with a steel disc and then joined with the acrylic resin block, which slid onto the parallel pins. The mandibular impression tray could be inserted into the patient's mouth in one piece because the acrylic resin block was elevated on the long pins, and the tray could be folded in the horizontal plane.

**Figure 3:**
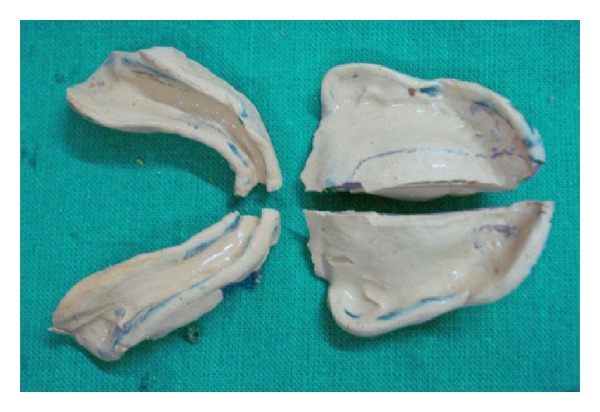
Final impression in sectional tray.

Border moulding was alternately done for the first and second halves of the sectional trays. Impression trays were inserted into the patient's mouth in two separate pieces: left and right and stabilized by means of the acrylic resin block. Final impressions were made by using zinc-oxide eugenol impression paste (DPI impression paste, DPI, India) in sectional trays, which were stabilized intraorally with acrylic resin block. After the impression paste set, the acrylic resin blocks were detached in the mouth, and the right and left pieces were removed separately by fracturing the impression material. The acrylic resin blocks were carefully joined out of the mouth, and after it was determined that the fracture line joined smoothly, dental stone was poured (Figure 3).

**Figure 4:**
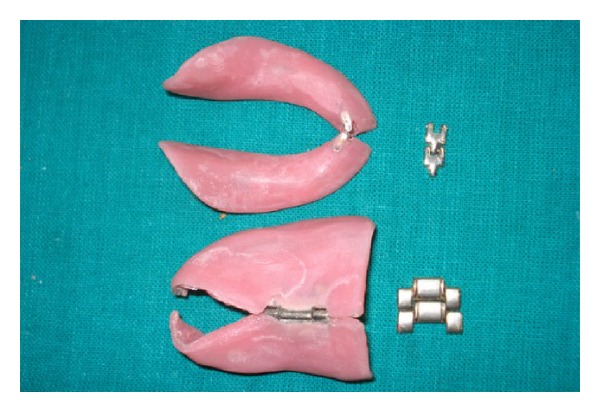
Temporary denture base with hinge.

The maxillary and mandibular denture bases were prepared in two pieces: right and left. These pieces were joined by overlapping one on the other by 2 mm in the midline. A stainless steel hinge was fitted with autopolymerizing acrylic resin in the centre of the axis connecting the denture bases (Figure 4).

Jaw relation record was obtained with the use of occlusion rims oriented to the established vertical dimension of occlusion, the anatomic occlusal plane, and the patient's centric relation. The try-in sectional denture was evaluated to verify jaw relations and tooth arrangement.

**Figure 5:**
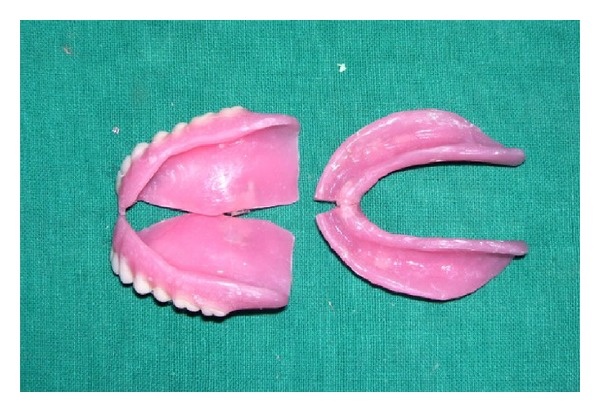
Foldable complete denture.

Heat cure acrylization was carried out alternately for right and left halves of the denture bases, and to prevent flow of resin into the connecting area, silicone impression material was placed into the gap in the hinge design. The denture was deflasked, trimmed, and polished (Figure 5).

Home care instructions (oral hygiene instruction, insertion, and removal of prosthesis) were and imparted to the patient, routine followup appointments were scheduled.

## 4. Discussion

Many authors have advised sectional custom trays and collapsible denture systems with complicated attachment devices, for example, locking levers (various pins, bolts, and Lego pieces), [3] hinges, [4, 5] orthodontic expansion screws, magnet systems, and so forth. For the patient described here, 4 parallel pins and an acrylic resin block fitted on these pins serve as a locking mechanism.

The use of different size pins in the mandibular impression tray made it possible for the tray to be folded in the horizontal plane and inserted in one piece, facilitating impression procedure. It was believed that the cross-section of the mandibular impression paste was not wide enough in the midline and that this would negatively affect the stability of the right and the left tray pieces. Thus, the pins on the mandibular tray were arranged in 2 different planes, and the resin block fitted on these pins ensured the proper approximation of two halves of the tray.

When the oral opening is limited, joining the pieces of a sectional denture base intraorally may be problematic. For this reason, we preferred to fabricate the collapsible (foldable) design of maxillary and mandibular complete denture.

## 5. Summary and Conclusion

**Figure 6:**
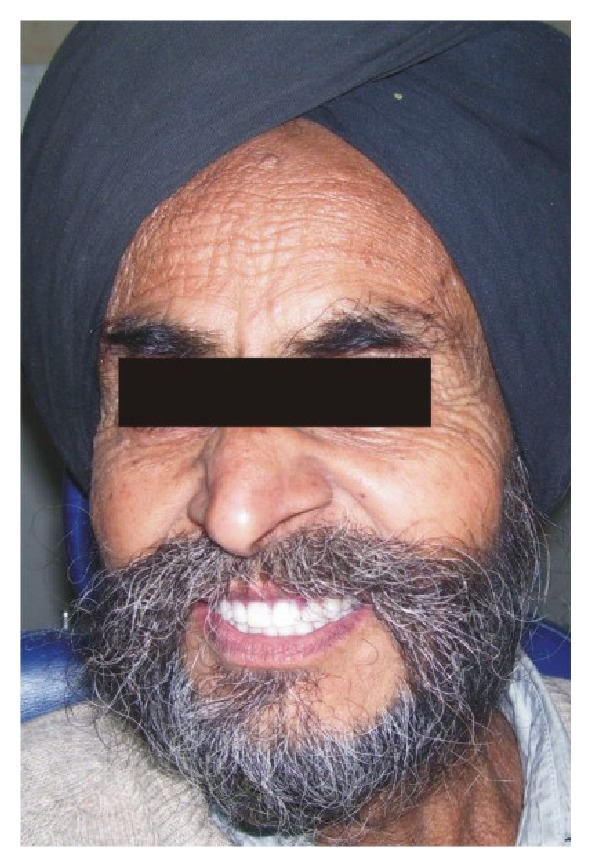
Postoperative photograph.

Severe reduction of oral opening renders access to the oral cavity difficult for dental procedures. This paper describes the impression procedure for a patient with restricted mouth opening using a sectional impression tray and fabrication of sectional maxillary and mandibular denture.  Figure 6 presents a patient who has been wearing such appliances successfully for the past 2 years.
